# The Claim of the Doctor

**Published:** 1916-03-25

**Authors:** 


### The Hospital

The Workers' Newspaper of Administrative Medicine and Institutional Life, Administration, National Insurance and Health.

No. 1554, Vol. LIX. SATURDAY, MARCH 25, 1916. PRICE ONE PENNY.

### THE CLAIM OF THE DOCTOR.

It is a commonplace to say that success in many of the relations established between a man and his fellows is determined by mutual sympathies and comprehensions rather than by formal rules and definitions; and the more intimate the relations the more appropriate is this statement. Regulations may be essential for those who work in combination for a common purpose, but who will venture to frame in a form of words the loyalties of friendship? There are, indeed, phases of life, and these by no means the least valuable, where it may be said truly that the letter killeth and only the spirit giveth life. In regions of this order a statement of law and rule, even if possible, would be largely useless. For those who have the root of the matter in them such a prescription is unnecessary, while for the rest it would be of no avail. It is on a sense of things which defies definition that the ordering of life to gracious ends very largely depends. Yet there remains the necessity of the translation of feeling into action, and this may perchance be clumsy, however willing the spirit. True that charity of interpretation and insight will see the motive in spite of the blunder, but accumulated strain even here may organise disaster. Hence, for the intimacies of life, though rules may not be defined, some reflections on principles are advisable and a study of situations is profitable or even essential. These considerations may perhaps to some seem rather high-flown when applied to the mutual relations of doctor and patient. Personally we do not think so, for this relation is not only an intimate one, but, at its best, is a possible contribution to the things which stand secure and which help to redeem life from poverty and narrowness of spirit. Of course, we are thinking here not so much of a chance and passing association—though here, also, the ideal is not beyond reach—but rather of the more sustained and prolonged relations which arise from continued and repeated opportunities for confidence on the one side and for help on the other, though in phrasing the position in these terms we are far from implying anything but a mutual benefit. This, indeed, is the essence of our claim—that in such relations rightly conducted service is twice blessed.

Now, when relations between doctor and patient are discussed by the laity it is the duties and obligations and responsibilities of the doctor which very naturally take the most prominent place. To say that in this judgment the sole responsibility of the patient is the payment of a fee would be an exaggeration. Yet, truth to tell, the code, so far as it is expressed, often hardly advances beyond this beggarly element. We have no desire to minimise the importance of this part of the bond, for the artist must be paid, not because the money is an apt reward for his work, but because, being a man, he needs must live. But we do wish to say that if there are to be good patients, even as good doctors, there is something due from the lay side other than the mere payment of a fee. Perhaps, at least in theory, everyone will allow us to add that a further essential condition is obedience to orders, though a curious freakish tendency to self-congratulation when these are defied without apparent penalty is not unknown, just as if winning tricks were proof of good play. To prompt payment of just debts and to honest adherence to accepted advice we may reasonably add as claims on the patient some thoughtful consideration for the doctor's time, health, and convenience, for he, too, is "subject to the same diseases, healed by the same means, warmed and cooled by the same winter and summer" as are the rest of mankind. If for himself the patient wishes consideration in these respects he may properly be asked to practise the same virtues towards his medical adviser.

What has just been said, however, relates largely to what may be called the mechanical side of the subject which we here discuss, and it may be doubted whether it is from these directions that failure in the relations between doctor and patient often arrives. There is, widely we are sure, patience and forbearance both on one side and on the other. The more serious blows arise from an entirely different source, and the patient who inflicts them is often quite ignorant of the pain he produces. Our reference is to actions which in the doctor's eyes, can only mean a failure of loyalty and confidence. The position arises when wisely or unwisely the patient thinks that he would like to have guidance other than that provided by his usual medical attendant. This may be quite a reasonable demand, and in any event it is a request or suggestion to which no sensible adviser will turn a deaf ear. Even if he judges the proposal unnecessary or unreasonable he will recognise it as an important fact in the situation, and one not to be dismissed without reason or discussion. The problems of medical practice are not invariably of such simplicity that they may be more readily solved by one head than by two, and, put at the lowest, the matter is of high importance to the patient and he is quite entitled to any help which he fancies will give him confidence in the issue. What a personal or family medical adviser objects to is the cultivation of such further advice without previous consultation with himself. Here, above all other things, is the stone of stumbling and the rock of offence. For such a proceeding implies that on a point which concerns the patient's health the patient did not think it worth while to consult his usual adviser; and it is not in human nature to suffer such treatment gladly. Few things, indeed, are more galling to a doctor who has carefully studied his patient than to receive a letter from some practitioner of consulting rank telling him what he already knows perfectly well. Such experiences dry up the wells of sympathy and harden relations which, in order to be most helpful, ought to be full of confidence and regard. That a patient may change his medical adviser as often as he pleases is not in dispute, nor is there any question that he may ask his doctor to discuss the position with any other reputable medical man. These things are open and above-board, and no one has a right to take offence at them. But the action here contemplated has not these qualities. On the contrary, it is wanting in frankness and loyalty, and where it is practised it is hopeless to expect that a medical adviser can either lend his full sympathy or make his best efforts. Too often in this way, and to the hurt alike of doctor and patient, is spoiled a relationship which, unchecked, has the promise of high influence and mutually helpful discourse.

